# A Combined Cyto- and Histopathological Diagnostic Approach Reduces Time to Diagnosis and Time to Therapy in First Manifestation of Metastatic Spinal Disease: A Cohort Study

**DOI:** 10.3390/cancers16091659

**Published:** 2024-04-25

**Authors:** Leon-Gordian Leonhardt, Annika Heuer, Martin Stangenberg, Malte Schroeder, Gabriel Schmidt, Lutz Welker, Gunhild von Amsberg, André Strahl, Lara Krüger, Marc Dreimann, Carsten Bokemeyer, Lennart Viezens, Anne Marie Asemissen

**Affiliations:** 1 Division of Spine Surgery, Department of Trauma and Orthopaedic Surgery, University Medical Center Hamburg-Eppendorf, 20251 Hamburg, Germany; 2 Department of Spine and Neurosurgery, Hospital Tabea Hamburg, 22587 Hamburg, Germany; 3 Institute of Pathology with the Sections Molecular Pathology and Cytopathology, University Medical Center Hamburg-Eppendorf, 20251 Hamburg, Germany; 4 IInd Medical Clinic and Policlinic for Oncology, Hematology, Bone Marrow Transplantation with Department of Pneumology, University Medical Center Hamburg-Eppendorf, 20251 Hamburg, Germany; 5 Division of Orthopaedics, Department of Trauma and Orthopaedic Surgery, University Medical Center Hamburg-Eppendorf, 20251 Hamburg, Germany; 6 Center for Spine Surgery, Clinic for Neuroorthopedics and Spinal Cord Injuries, Orthopedic Clinic Markgröningen gGmbH, 71706 Markgröningen, Germany

**Keywords:** spinal neoplasms, cytopathology, delayed diagnosis, time to treatment, surgery

## Abstract

**Simple Summary:**

Symptomatic spinal lesions often indicate the presence of advanced malignant tumors. Alongside a surgical intervention for spinal treatment, it is imperative to confirm the diagnosis to devise further therapy strategies. Unfortunately, confirming the diagnosis through histopathology (HP) can take up to 14 days. However, cytopathology (CP) can offer an initial assessment within a few days. This study examines the impact of a simultaneous CP analysis alongside HP during spinal surgery on the time to diagnosis (TTD) and the time to the first tumor-specific therapy (TTT) in patients undergoing spinal surgery for suspected spinal malignancies. The findings demonstrate that concurrent CP significantly reduces both the TTD and TTT. Hence, it is recommended to incorporate CP alongside HP as a standard procedure in the surgical management of suspected malignant spinal lesions.

**Abstract:**

Malignant spinal lesions (MSLs) are frequently the first manifestation of malignant disease. Spinal care, diagnostic evaluation, and the initiation of systemic therapy are crucial for outcomes in patients (pts) with advanced cancer. However, histopathology (HP) may be time consuming. The additional evaluation of spinal lesions using cytopathology (CP) has the potential to reduce the time to diagnosis (TTD) and time to therapy (TTT). CP and HP specimens from spinal lesions were evaluated in parallel in 61 pts (CP/HP group). Furthermore, 139 pts in whom only HP was performed were analyzed (HP group). We analyzed the TTD of CP and HP within the CP/HP group. Furthermore, we compared the TTD and TTT between the groups. The mean TTD in CP was 1.7 ± 1.7 days (d) and 8.4 ± 3.6 d in HP (*p* < 0.001). In 13 pts in the CP/HP group (24.1%), specific therapy was initiated based on the CP findings in combination with imaging and biomarker results before completion of HP. The mean TTT in the CP/HP group was 21.0 ± 15.8 d and was significantly shorter compared to the HP group (28.6 ± 23.3 d) (*p* = 0.034). Concurrent CP for MSLs significantly reduces the TTD and TTT. As a result, incorporating concurrent CP for analyzing spinal lesions suspected of malignancy might have the potential to enhance pts’ quality of life and prognosis in advanced cancer. Therefore, we recommend implementing CP as a standard procedure for the evaluation of MSLs.

## 1. Introduction

Malignant spinal lesions (MSLs) are a common first manifestation of malignant disease in cancer patients (pts) [1,2]. The five most frequent malignancies that present with spinal manifestations are multiple myeloma, lung cancer, breast cancer, renal cancer, and prostate cancer [3,4,5,6,7]. Most pts present with advanced and often terminal disease. Besides the management of local symptoms such as back pain and neurological deficits, the confirmation of diagnosis, staging, assessment of prognosis, and induction of systemic therapy are crucial for the outcome and optimization of palliative care.

Nevertheless, the overall prognosis of advanced cancer has improved substantially over the last decades [8,9]. In contrast, a delay in therapeutic management worsens the morbidity and mortality for advanced cancer [10]. Therefore, a prompt initiation of systemic cancer therapy is mandatory to also preserve optimal outcomes in advanced disease.

Time to therapy initiation (TTT) is a pivotal challenge when treating advanced cancer pts in a multidisciplinary spinal oncology team. The time to diagnosis (TTD) is a critical factor influencing the TTT, especially since advanced diagnostic methods are frequently time consuming. In addition, sufficient tissue sampling is essential for a histopathological (HP) diagnosis to make a reliable diagnosis [11]. While a bone biopsy with immunohistochemistry (IHC) is easy, cost effective, and represents the gold standard [12,13], the necessary decalcification of specimens before diagnostic procedures takes up to 10 work days (d) [14]. In contrast, a cytopathology (CP) diagnosis from bone marrow aspirations using the Jamshidi procedure or imprint cytology (IC) from specimens can be obtained within a few hours. Furthermore, most IHC methods and molecular genetics can be performed in CP samples [15,16,17].

Recently, we presented a diagnostic approach for evaluating MSLs by conducting CP and HP simultaneously using intralesional spinal aspiration cytology (ISAC), demonstrating a high level of adequacy and concordance between CP and HP [18]. Moreover, ISAC is easy to perform intraoperatively and can be implemented easily into an intraoperative workflow [18]. Furthermore, in hematological malignancies, in addition to the conventional morphology, it is mandatory to obtain material for further diagnostic procedures for the assessment of prognostic factors including cytogenetics, molecular pathology, or flow cytometry [19].

In this trial, we provide an update on the diagnostic value of intraoperative CP complementary to HP in a larger group of pts. However, the focus of this work is on the comparative analysis of the TTD and TTT in advanced cancer pts with spinal metastases in whom HP was performed alone or in parallel with CP.

## 2. Materials and Methods

This study was reported to the local ethics committee (ethics vote: 2023-300403-WF). Written informed consent was waived by the local ethics board. The data collection and evaluation were retrospective and anonymized.

In this study, we analyzed the sensitivity and specificity of CP and concordance with HP (CP/HP group). Furthermore, we analyzed the TTD and TTT in the CP/HP group in comparison with a patient cohort who underwent surgery for MSLs of a primarily unknown tumor disease and were diagnosed using HP alone (HP group). A retrospective collection and analysis of the data was carried out in both study groups. Pts with previously diagnosed cancer were not eligible for participation in the analysis. A follow-up was performed until the initiation of oncological system therapy or radiation.

All pts aged 18 years or older who had initial manifestations of spinal bone lesions radiologically classified as suspicious for malignancy, no primary tumor or other metastatic lesions to be biopsied, or surgical indication for spinal metastasis necessitating the procedure despite other metastases were eligible for the CP/HP study group. In addition to primary spinal imaging using computed tomography (CT) and magnetic resonance imaging (MRI), a staging CT was performed, and a panel of biomarkers was determined to further narrow the search for the primaries. The biomarker panel included the following markers: total PSA, free PSA, CA15-3, CA19-9, CEA, CA125, NSE, AFP, S100, clonal protein, M-gradient, IgG, IgA, IgM, free light chain lambda, free light chain kappa, and free light chain quotient.

For the HP group, we analyzed all pts who underwent surgery for MSLs with a primarily unknown tumor in our clinic between January 2016 and December 2020 before the implementation of additional cytopathology (CP) analysis. In this patient cohort, the TTD and TTT in terms of the initiation of oncological system therapy or radiation were analyzed.

Sample collection in the CP/HP group was performed as recently described in detail [18]. In brief, the CP samples were obtained during spinal surgery in addition to HP using ISAC through material aspiration using a Jamshidi needle. If ISAC could not be performed due to dry tap aspiration, IC was prepared by dabbing the obtained tissue specimen on a glass slide.

The slides prepared for the CP analysis were stained using standard protocols such as hematoxylin and eosin (HE) or May Gruenwald and Giemsa (MGG). IHC was not regularly performed on cytology samples due to institutional standards. The HP samples were processed according to standard procedures, including extended IHC staining protocols for any previously undiagnosed malignant disease for diagnostic and prognostic information. In brief, after fixation in formalin, decalcification in EDTA was performed, followed by embedding in paraffin. After cutting, HE and IHC stainings were performed. The samples for CP and HP were analyzed separately by separate teams as part of the clinical standard.

A statistical analysis was performed using SPSS 27.0 (IBM Corp., Armonk, NY, USA). For the statistical analysis, we analyzed the diagnostic value of ISAC, focusing on the sensitivity, specificity, positive predictive, and negative predictive values. The concordance of HP and CP was demonstrated by determining Cohen’s kappa. A comparison of the groups with respect to the TTD and TTT was performed using two-tailed *t*-test. Fisher’s exact tests were applied for dichotomous categorial variables. The significance level was defined as *p* < 0.05.

## 3. Results

After the establishment of ISAC, we extended the already published population of 44 pts [18] in the diagnostic routine procedure up to 61 pts, and now, we additionally evaluated the pts for parameters of clinical oncology, especially the TTD and TTT.

### 3.1. Patient Characteristics

Between February 2021 and January 2022, we extended the cohort and included 61 pts in the CP/HP group. All pts underwent emergency or urgent spinal surgery for MSLs. Of these 61 pts, 23 were female (37.7%). The median age of all pts was 65 (20–86) years (y). The HP results showed a diagnosis of malignancy in 55 cases (90.2%), whereas 6 pts had a non-malignant disease.

The four most common malignant diagnoses in the CP/HP group were multiple myeloma (14/55, 25.5%), breast cancer (9/55, 16.4%), adenocarcinoma of the lung (8/55, 14.5%), and prostate cancer (5/55, 9.1%), covering 36/55 (65.5%) of all malignant diagnoses.

In the CP/HP group, the HP analysis was inconclusive in three cases. The first patient had adenocarcinoma of unknown primary tumor with bone, liver, and lung metastasis. Imaging or endoscopy could not identify a primary tumor. In the second patient, HP revealed an undifferentiated carcinoma with high PD-L1 expression and a high TPS. Imaging suggested urothelial carcinoma with a suspicious primary tumor in the right bladder dome. A systemic treatment with anti-PD-L1-directed pembrolizumab was initiated. The third patient had a TTF-negative, undifferentiated adenocarcinoma but CT imaging presented a spiculated pulmonary lesion in the left lower lobe. He was an active smoker. In consideration of all findings, the diagnosis of bronchopulmonary adenocarcinoma was established.

For the HP group, a total of 583 consecutive pts who underwent spinal surgery for MSLs were identified. Among this cohort, 139 pts who had not yet received a cancer diagnosis were included in the HP group. The median age was 67 (21–85) y. In total, 55 pts were female (39.6%). The four most common malignant diagnoses were comparable to the CP/HP group, with multiple myeloma (28/139, 20.1%), adenocarcinoma of the lung (21/139, 15.1%), breast cancer (11/139, 7.9%), and prostate cancer (10/139, 7.1%) representing 70/139 (50.4%) of all malignant diagnoses here. The patient characteristics are summarized below (Table 1).

There were no significant differences between the CP/HP group and the HP group in terms of demographics and disease characteristics. Therefore, the two groups are considered comparable. The details of this analysis can be found in Table S1.

A detailed list of all histological diagnoses is presented below (Table 2).

### 3.2. Feasibility of Simultaneous Cytology from the Spinal Lesion and Bone Marrow

The CP specimens were sufficiently obtained intraoperatively using ISAC or IC in 57 of the 61 pts of the CP/HP group (93.4%). No patient suffered complications associated with the additional diagnostic procedure, especially an elongation of the operation time, blood loss, infection, or neurological deficits. In pts who underwent surgery in prone position, additional iliac crest bone marrow aspiration cytology test was obtained for the completion of staging following the potential diagnosis of hematologic neoplasms and to obtain sufficient bone marrow material for a flow cytometric, cytogenetic, or molecular genetic diagnosis.

In four pts (6.6%), no sufficient CP could be performed. A “dry tap” phenomenon was encountered in three cases (4.9%). All these pts had plasma cell myeloma. The concurrently performed bone marrow biopsy from the iliac crest provided the diagnosis of multiple myeloma in two cases, while one case showed no sign of malignancy in the iliac crest. In this case, spinal HP revealed solitary myeloma. In one case, the wrong anticoagulant (heparin instead of citrate) was used for the ISAC and biopsy of the iliac crest, rendering the sample unsuitable for evaluation. The spinal HP revealed multiple myeloma.

### 3.3. Diagnostic Accuracy of Cytology and Concordance with Histology

The CP enabled the correct identification of malignant lesions in 49 pts; furthermore, no malignant finding was reported in 5 pts, resulting in a concordance of CP and HP morphological diagnosis in 54 of the 57 (94.7%) pts, with a Cohen’s kappa of 0.74 (strong concordance) according to categories of malignant versus no malignant finding. Malignant cells in CP were further described as lymphoma, malignant plasma cells, atypical cells, highly suspected for malignancy, adenocarcinoma cells, small cell cancer cells, and melanoma cells. To identify MSLs, CP presented a sensitivity of 0.96 and a specificity of 0.83, respectively. The positive predictive value in CP was 0.98, and the negative predictive value of CP was 0.71. In one patient, a diagnosis of multiple myeloma was made in the CP specimen from the MSL, which could not be detected in the HP sample. The diagnosis was confirmed by the bone marrow aspiration from the iliac crest and the detection of a significant monoclonal paraprotein in serum protein electrophoresis. A second histopathological examination of the MSL performed during the course also confirmed the diagnosis of multiple myeloma.

The CP diagnoses were multiple myeloma, lymphoma, non-hematopoietic malignant cells (solid malignancy including adenocarcinoma, small cell carcinoma, and melanoma), and the absence of malignant cells. Related to these categories, CP enabled correct differentiation in 54 of the 57 cases (94.7%) with a Cohen’s kappa of 0.89, demonstrating a very high concordance. Table 3 shows the concordance of differentiation of spinal lesions by CP in reference to HP in a cross-tabulation (Table 3).

The exact diagnosis was made by further IHC staining of the HP specimens in addition to imaging and biomarkers.

### 3.4. Time to Diagnosis (TTD) for CP/HP and HP

**Figure 1:**
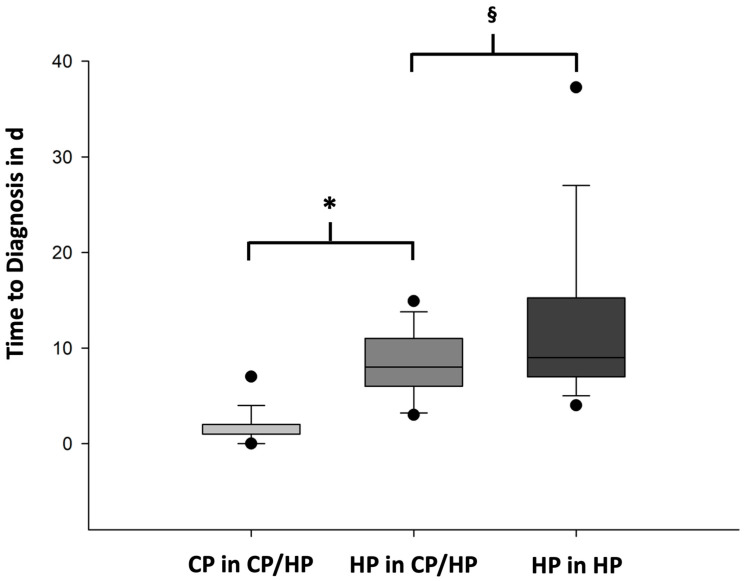
Time to diagnosis of CP vs. HP within CP/HP group and HP in the CP/HP group vs. HP group. Data are shown in boxplots. Boxes indicate the upper and lower quartiles, the line indicates the median, the upper and lower whiskers indicate the 1.5× interquartile range, and black dots indicate the 5% and 95% percentiles. Data from the comparative analysis of the time to diagnosis (TTD) in days (d) of cytopathology (CP, light gray) and histopathology (HP, mid gray) within the CP/HP group. CP in CP/HP group (*n* = 57) vs. HP in CP/HP group (*n* = 61), * *p* < 0.001 (*t*-test). Furthermore, data from the comparative analysis of the TTD in d of HP in HP group (dark gray). HP in CP/HP group (*n* = 61) vs. HP in HP group (*n* = 139), ^§^ *p* < 0.001 (*t*-test).

We analyzed the TTD of CP and HP within the CP/HP group and in comparison to the HP group. The mean TTD in CP was 1.7 ± 1.7 d. The mean TTD in HP including the IHC analysis was 8.4 ± 3.6 d in the CP/HP group. The TTD was significantly shorter in CP compared to HP (*p* < 0.001) (Figure 1). In the HP group, the mean TTD was 13.1 ± 10.1 d, which was significantly longer compared to the TTD of HP in the CP/HP group (*p* < 0.001) (Figure 1).

### 3.5. TTT in the CP/HP Group Compared to the HP Group

**Figure 2:**
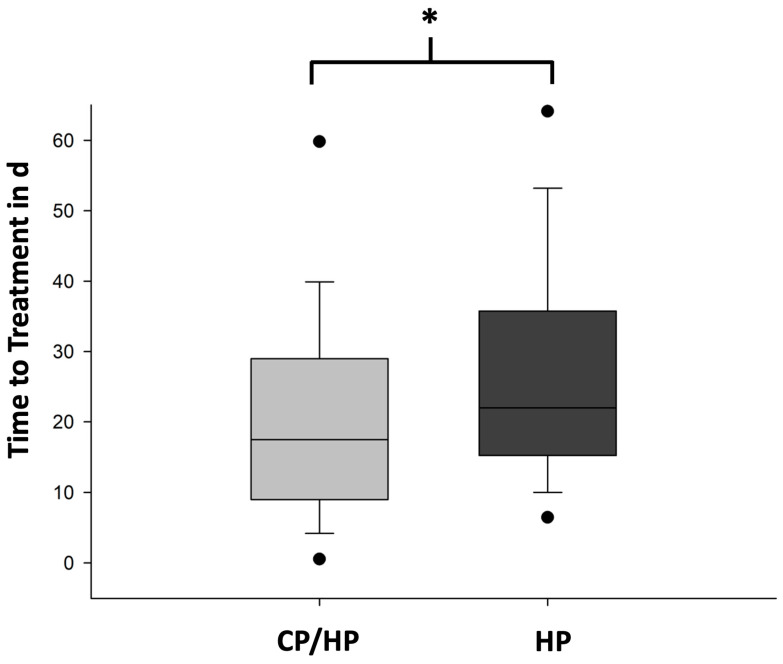
Time to therapy in CP/HP group vs. HP group. Data are shown in boxplots. Boxes indicate the upper and lower quartiles, the line indicates the median, the upper and lower whiskers indicate the 1.5× interquartile range, and black dots indicate the 5% and 95% percentiles. Data from the comparative analysis of the time to therapy (TTT) in days (d) of the cytopathology/histopathology (CP/HP) group (light gray) and the histopathology (HP) group (dark gray) are shown here. The TTT in CP/HP group (*n* = 50) vs. the TTT in HP group (*n* = 128), * *p* = 0.034 (*t*-test).

Furthermore, the TTT was defined as the time from surgery to the first dose of systemic oncological treatment or radiation. The mean TTT was 21.0 ± 15.8 d (*n* = 50) in the CP/HP group and 28.6 ± 23.3 d (*n* = 128) in the HP group (*p* = 0.034) (Figure 2).

In 13 of the 55 pts diagnosed with MSLs in the CP/HP group (24.1%), a specific therapy was initiated based on the CP findings in combination with imaging and biomarker results before the completion of HP.

Of these 13 pts, 4 had myeloma, which was identified through CP findings in combination with significant monoclonal gammopathy. Steroid-free monotherapy with daratumumab was initiated in two pts with an indication for urgent therapy and a high risk of wound healing impairment only two d after surgery.

In four pts who received an early initiation of therapy, a diagnosis of prostate carcinoma was made, based on evidence of adenocarcinoma cells in CP in combination with an elevated PSA, CT imaging results, and the male sex. All pts showed osteoblastic and osteolytic lesions in parallel. In these pts, nonsteroidal anti-androgen treatment with bicalutamid was started before the initiation of GnRH analogue therapy.

Two pts were diagnosed with small cell lung carcinoma (SCLC). In one patient, a cytological diagnosis including IHC revealed the presence of a small cell carcinoma with expression of synaptophysin. CT imaging showed a mass in the lateral left upper lobe with contact with the pleura, confirming the diagnosis of SCLC. Treatment was initiated with a combination of chemotherapy, biologicals, and spinal radiation. In the second patient with SCLC, the diagnosis was made from the intraspinal soft tissue, which was available two d after surgery. CT imaging showed a large right pulmonary lesion, the NSE level was measured at 97.1 µg/L, and the LDH was significant. Therefore, the treatment for SCLC was started before receiving the HP results from spinal bone samples.

One patient with a history of melanoma 13 years prior was diagnosed with metastatic melanoma with osseous, hepatic, and cerebral manifestations. Systemic checkpoint inhibitor therapy was started before an HP diagnosis based on the detection of pigmented cells in CP. The S100 and LDH were significantly elevated.

A female patient was diagnosed with breast carcinoma with an exulcerating tumor in the left breast. Due to unstable spinal lesions, an emergent spinal surgery was indicated. CP of the spinal lesion revealed an adenocarcinoma. Concurrently, a punch biopsy of the suggested primary tumor was performed. The biopsy confirmed the diagnosis of an estrogen receptor-positive status, progesterone receptor-negative status, and Her2-negative status breast carcinoma. The tumor markers CEA and CA15-3 were elevated and measured at 86.0 µg/L and 458.9 kU/L, respectively. Before receiving the final HP results from spinal bone samples, a systemic anti-hormone therapy was initiated.

Additionally, one patient was diagnosed with carcinoma of biliopancreatic origin. In this patient, CP from the vertebral body revealed a solid malignant tumor. CT imaging was highly suspicious for cholangiocellular carcinoma, with poorly defined extensive hypodensity centrally in the right hepatic lobe and significant intrahepatic cholestasis. The CA19-9 level was measured at 54.9 kU/L. After an interdisciplinary case discussion at a spinal tumor conference, systemic chemotherapy utilizing cisplatin and gemcitabine and spinal radiation were started prior to receiving the final HP results.

Detailed patient characteristics and relevant biomarkers are shown below (Table 4).

Specific therapy was initiated at a mean of 4.5 ± 3.4 d before the HP results were received.

No patient in the CP/HP cohort experienced wound healing complications following the early initiation of therapy due to cytotoxic side effects.

In the CP/HP group, one patient (1.6%) did not receive tumor-specific systemic therapy and received the best supportive care after spinal surgery due to their limited performance status. The diagnosis for this patient was a bronchopulmonary adenocarcinoma. In contrast, in the HP group, 15 pts (10.8%) did not receive systemic therapy. All of them were diagnosed with a solid malignancy. Additionally, in the HP group, one patient (0.7%) passed away before the completion of the HP diagnosis due to advanced cancer.

## 4. Discussion

Concurrent CP in addition to HP in MSLs during spinal surgery is fast, easy, safe, and demonstrates a highly specific diagnostic reliability as well as a high concordance with final HP. The sensitivity for malignancy was extremely high in comparison to HP, whereas the specificity was lower. The high sensitivity of CP allows for the safe identification of MSLs. Thus, supplemental CP from malignant lesions could help to exclude malignancy in atraumatic spinal fractures, such as osteoporotic fractures, since sufficient material could be obtained by aspiration in all non-malignant lesions.

However, the “dry tap” phenomenon in MSLs can impede the sensitivity of malignancy detection and should always be correlated with results from HP. Based on our experience, aspirating material from MSLs resulted in an insufficient amount of examination material to conduct all necessary tests, especially in cases of multiple myeloma, primarily due to “packed cells”.

CP/HP correlation was already established in non-bone(-marrow) specimens for the assessment of quality [20]. A CP analysis is widely used for screening and management, e.g., in gynecology [21,22], bronchoscopy samples [23,24], testicular and hepatobiliary tumors [25,26], and thyroid carcinomas [27].

The combination of CP with HP proved to be highly beneficial in facilitating early patient assessment and management for individuals with MSLs. This allowed for the timely involvement of a multidisciplinary team, including medical oncologists and radiotherapists, as well as the planning of further diagnostic procedures or the prompt implementation of necessary medical interventions. Otherwise, pts with an uncomplicated follow-up after spinal surgery might be discharged without a definitive diagnosis, potentially leading to the delayed diagnosis of advanced cancer due to time-consuming decalcification procedures or missed follow-ups. The significantly shorter TTD in the CP/HP group may reduce this risk.

However, the workflow for collecting and processing samples can vary significantly depending on the institution’s setup. When a CP laboratory is located within the same institution, samples can be processed immediately. For hematological samples, additional specialized diagnostics like flow cytometry, genetics, and molecular therapy can also be performed promptly. However, if the CP laboratory is not conveniently located near the operating room (OR), the samples may need to be initially processed by the OR staff, particularly during nighttime or on weekends. This situation can introduce delays in the processing of samples.

Although most diagnostic procedures are standardized for HP and IHC [15,16,28], due to recent advances in diagnostic procedures, a sufficient diagnosis can be made from small tissue samples and even a single cell analysis, including the development of a liquid biopsy. In addition, advanced diagnostic procedures such as next-generation sequencing (NGS) for comprehensive malignant diagnosis can also be performed on cytological material [17,29].

In the 61 pts examined, malignant disease was found to be the cause of the spinal lesion in 55 pts. No primary malignant bone tumor was identified. Most commonly, multiple myeloma (25.5%), breast cancer (16.9%), lung adenocarcinoma (14.6%), and prostate cancer (9.1%) were identified. These data are in line with those of other authors who also found predominantly advanced solid neoplasms [2]. These findings underscore that pts with MSLs due to advanced malignant disease have a high medical need for interprofessional and interdisciplinary therapeutic interventions from spinal surgery, medical oncology, and rehabilitative medicine.

The significantly shorter TTD of CP compared to HP allows for the early interdisciplinary focused, disease-related planning of diagnostic and treatment procedures. Furthermore, in the CP/HP group, the systemic treatment of malignant disease was started in 13 (24.1%) pts before receiving the results from HP. The diagnosis was made using CP together with clinical findings, biomarkers, and imaging results. In all 13 cases, we were able to show that the CP diagnosis was confirmed by HP results. Furthermore, 25.5% of the pts were diagnosed with multiple myeloma, which can be diagnosed by cytomorphology [30]. Therefore, we believe that spinal lesions suspected of malignancy without a previously known underlying malignancy should be analyzed in parallel with CP utilizing ISAC or IC in addition to HP.

Moreover, for pts with multiple myeloma who need further sampling for a molecular genetics analysis and flow cytometry, it is comfortable to undergo a bone marrow puncture under general anesthesia during spinal surgery.

Furthermore, the TTD for HP was shorter in the CP/HP group compared with the retrospective HP patient cohort, suggesting that early interdisciplinary communication also accelerates the pathology workflow. In addition, the shortening of the TTD was correlated to an earlier initiation of systemic treatment.

Another principal aspect influencing the TTT is the postoperative recovery time of the pts. In the current study, the postoperative recovery time of the CP/HP and HP groups does not appear to be different from a surgical point of view, with clinically comparable groups and the same internal hospital therapy standards. In our opinion, the differences in the TTT are attributed to the organizational factor of a shorter TTD.

The timely availability of a malignant diagnosis in CP allows for the initiation of prognosis-appropriate diagnostic, supportive, and therapeutic interventions related to the pathology of the spine as well as the underlying oncological disease. This was supported by the finding that only 1.6% of the CP/HP group did not receive a systemic cancer therapy in contrast to 10.8% for the HP cohort. This may also be associated with the deterioration of the performance state during the delay of diagnostic procedures. The timely initiation of systemic therapy has the potential to reduce patient mortality and morbidity in pts with advanced cancer.

The limitations of this study are the retrospective study character and the selection of pts and lesions due to the sole enrollment of pts with spinal lesions suspicious of malignancy and an indication for surgical intervention.

## 5. Conclusions

The evaluation of CP from MSLs in conjunction with clinical parameters enables a shorter TTD compared to HP alone. This allows for the rapid planning of further diagnostics and therapies, which shortens the TTT. A faster initiation of therapy could improve the quality of life and prognosis of pts with advanced cancer. The early introduction of a multidisciplinary oncology team is mandatory for the optimal management of pts with advanced cancer presenting with spinal lesions.

## Figures and Tables

**Table 1:** Patient characteristics.

	CP/HP Group	HP Group
Number of patients	61	139
Sex	37.7% female	36.9% female
Age	65 (20–86) years	67 (21–85) years
Anatomic region		
• Cervical spine	3	35
• Thoracic spine	31	74
• Lumbar spine	23	44
• Pelvis	7	8

CP—cytopathology; HP—histopathology.

**Table 2:** Malignant diagnoses.

Diagnosis	N (CP/HP Group)	N (HP Group)
Multiple myeloma	14	28
Breast cancer	9	11
Bronchopulmonal adenocarcinoma	8	21
Prostate cancer	5	10
Undifferentiated carcinoma	3	10
Small cellular lung cancer	3	5
Hepatocellular carcinoma	3	0
Renal cell carcinoma	2	7
Malignant melanoma	2	1
Squamous cell carcinoma of the lung	1	7
Cholangiocellular carcinoma	1	3
Malignant fibrous tumor	1	1
Epithelioid angiosarcoma	1	1
B-cell lymphoma	1	8
Dedifferentiated liposarcoma	1	0
Undifferentiated squamous cell carcinoma	0	9
Gastrointestinal adenocarcinoma	0	8
Thyroid cancer	0	1
Urothelial carcinoma	0	4
Ewing sarcoma	0	4
Non-malignant	6	0

CP—cytopathology; HP—histopathology; N—number.

**Table 3:** Concordance of cytopathology and histopathology in the CP/HP collective.

			Histopathology			
		Myeloma	Solid	Lymphoma	Non-Malignant	Total
	Myeloma	9	0	0	1	10
**Cytopathology**	Solid	0	39	0	0	39
	Lymphoma	0	0	1	0	1
	Non-malignant	1	1	0	5	7
	Total	10	40	1	6	57

CP—cytopathology; HP—histopathology.

**Table 4:** Patients in whom specific therapy was initiated prior to final HP from spinal lesion.

TTT in d	T before HP in d	Diagnose	Type of T	Relevant Biomarkers
0	6	Prostate cancer	Anti-androgen therapy	Total PSA: 4257.22 µg/L Free PSA: 14.07 µg/L
1	12	Prostate cancer	Anti-androgen therapy	Total PSA: 2625.07 µg/L Free PSA: 320.91 µg/L
7	1	Prostate cancer	Anti-androgen therapy	Total PSA: 248.86 µg/L Free PSA: 21.23 µg/L
6	7	Prostate cancer	Anti-androgen therapy	Total PSA: 1345.52 µg/L Free PSA: n.a.
0	9	Multiple myeloma	Chemo-, biological, steroid therapy	M gradient: 39.4 Clonal protein: 43.7 g/L IgG: 57.55 g/L IgA: <0.15 g/L IgM: 0.13 g/L Free light chain kappa: 4.92 g/L Free light chain lambda: 0.12 g/L Free light chain quotient: 41.00 g/L
4	7	Multiple myeloma	Steroid therapy	M gradient: 37.3 Clonal protein: 27.2 g/L IgG: 32.40 g/L IgA: 0.41 g/L IgM: 0.22 g/L Free light chain kappa: 0.017 g/L Free light chain lambda: 0.66 g/L Free light chain quotient: 0.03 g/L
4	3	Multiple myeloma	Anti-CD38 antibody	M gradient: 53.4 Clonal protein: 53.9 g/L IgG: 58.68 g/L IgA: 0.68 g/L IgM: 0.12 g/L Free light chain kappa: 0.5 g/L Free light chain lambda: 15.80 g/L Free light chain quotient: 0.03 g/L
7	2	Multiple myeloma	Anti-CD38 antibody	M gradient: n.a. Clonal protein: n.a. IgG: n.a. IgA: n.a. IgM: n.a. Free light chain kappa: 0.51 g/L Free light chain lambda: 1.39 g/L Free light chain quotient: 0.36 g/L
11	3	Small cellular lung cancer	Radiation, chemotherapy, checkpoint inhibitor	Total PSA: 0.88 µg/L Free PSA: 0.21 µg/L NSE: 97.1 µg/L
14	1	Small cellular lung cancer	Radiation, chemotherapy, checkpoint inhibitor	NSE: 14.5 µg/L
11	4	Melanoma	Dual checkpointinhibitors	n.a.
9	3	Breast cancer	Anti-hormone therapy	CA15-3: 458.9 kU/L CEA: 86.0 kU/L
9	1	Biliopancreatic carcinoma	Radiation	CA19-9: 48.1 kU/L CA125: 0.8 kU/L

HP—histopathology; T—therapy; TTT—time to therapy; d—days.

## Data Availability

The data presented in this study are available within this article. All data generated or analyzed during this study are included in this published article.

## Supplementary Materials

The following supporting information can be downloaded at: https://www.mdpi.com/article/10.3390/cancers16091659/s1, Table S1: Demographic and disease characteristics of the overall sample, the CP/HP- and HP group.
