# Supplementary material for: A Combined Cyto- and Histopathological Diagnostic Approach Reduces Time to Diagnosis and Time to Therapy in First Manifestation of Metastatic Spinal Disease: A Cohort Study
Source: Cancers (Basel). 2024 Apr 25;16(9):1659. doi: 10.3390/cancers16091659 (PMC11083103; doi:10.3390/cancers16091659)
Supplement: Supplementary file 1 [file cancers-16-01659-s001.zip › cancers-2945406-supplementary.pdf]

Table S1. Demographic and disease characteristics of the overall sample, the CP/HP- and HP group.

	**Overall (n=200)**	**CP/HP group (n=61)**	**HP group (n=139)**	***p*-value**
Age in years				
Mean (SD)	65.3 (12.5)	64.4 (13.6)	65.7 (12.1)	
Median, min-max	66, 21-86	65, 20-86	67, 21-85	0.497
Sex (n, %)				
Male	78, 39.0%	38, 62.3%	84, 60.4%	
Female	122, 61.0%	23, 37.7%	55, 39.6%	0.804
Time to diagnosis (TTD)				
Mean (SD)	11.7 (8.9)	8.5 (3.6)	13.1 (10.1)	
Median, min-max	9, 0-63	8, 2-16	9, 0-63	<0.001
Time to tumor-specific therapy (TTT)				
Mean (SD)	26.5 (21.7)	21.0 (15.8)	28.6 (23.3)	
Median, min-max	21, 0-177	17.5, 0-73	22, 0-177	0.034
Solid malignancy (n, %)	145, 72.5%	40, 65.6%	105, 75.5%	0.146
Hemic malignancy (n, %)	50, 25.0%	14, 22.9%	36, 25.9%	0.658
Prostate cancer (n, %)	15, 7.5%	5, 8.2%	10, 7.2%	0.804
Breast cancer (n, %)	20, 10.0%	9, 14.8%	11, 7.9%	0.138
Bronchopulmonal adenocarcinoma (n, %)	29, 14.5%	8, 13.1%	21, 15.1%	0.712
Multiple myeloma, (n, %)	42, 21.0%	14, 23.0%	28, 20.1%	0.654
Undifferentiated carcinoma (n, %)	13, 6.5%	3, 4.9%	10, 7.2%	0.548

CP - Cytopathology, HP – Histopathology, SD - standard deviation, min - minimum, max – maximum, TTD - Time to Diagnosis, TTT - Time to Treatment.
